# Expanding the boundaries of atomic spectroscopy at the single-cell level: critical review of SP-ICP-MS, LIBS and LA-ICP-MS advances for the elemental analysis of tissues and single cells

**DOI:** 10.1007/s00216-023-04721-8

**Published:** 2023-05-10

**Authors:** Claire Davison, Dany Beste, Melanie Bailey, Mónica Felipe-Sotelo

**Affiliations:** 1 School of Chemistry and Chemical Engineering, Faculty of Engineering and Physical Sciences, University of Surrey, Guildford, UK; 2 Department of Microbial Science, Faculty ofHealth and Medical Sciences, University of Surrey, Guildford, UK

**Keywords:** Single cell, Atomic spectroscopy, Laser ablation, Mass spectrometry

## Abstract

Metals have a fundamental role in microbiology, and accurate methods are needed for their identification and quantification. The inability to assess cellular heterogeneity is considered an impediment to the successful treatment of different diseases. Unlike bulk approaches, single-cell analysis allows elemental heterogeneity across genetically identical populations to be related to specific biological events and to the effectiveness of drugs. Single particle-inductively coupled plasma-mass spectrometry (SP-ICP-MS) can analyse single cells in suspension and measure this heterogeneity. Here we explore advances in instrumental design, compare mass analysers and discuss key parameters requiring optimisation. This review has identified that the effect of pre-treatment of cell suspensions and cell fixation approaches require further study and novel validation methods are needed as using bulk measurements is unsatisfactory. SP-ICP-MS has the advantage that a large number of cells can be analysed; however, it does not provide spatial information. Techniques based on laser ablation (LA) enable elemental mapping at the single-cell level, such as laser-induced breakdown spectroscopy (LIBS) and laser ablation-inductively coupled plasma-mass spectrometry (LA-ICP-MS). The sensitivity of commercial LIBS instruments restricts its use for sub-tissue applications; however, the capacity to analyse endogenous bulk components paired with developments in nano-LIBS technology shows great potential for cellular research. LA-ICP-MS offers high sensitivity for the direct analysis of single cells, but standardisation requires further development. The hyphenation of these trace elemental analysis techniques and their coupling with multi-omic technologies for single-cell analysis have enormous potential in answering fundamental biological questions.

## Background

### Metals and microbiology

Metal homeostasis within biological systems is critical for the immune response, metabolism and intracellular signalling [1]. Moreover, elevated and unregulated concentrations of certain elements have been linked to different diseases. Elevated concentrations of magnesium, chromium, zinc and silicon were found in tumour tissue compared with healthy tissues, identifying a diagnostic element fingerprint for colorectal biopsies [2]. An imbalance of iron, copper, zinc and calcium ions in brain tissue has been associated with the progression of Alzheimer’s disease [3], and elevated levels of iron and sodium in olfactory bulbs of Parkinson’s patients have been correlated with the loss of sense of smell [4]. For infectious diseases, the battle for metal ions is critical during host–pathogen interactions and has an important role in immunometabolism. Understanding metal acquisition and how metals are used by the host as bacteriostatic/bactericidal weapons has become an important focus in this field [5] as highlighted by the important role of copper, zinc and iron in the immune system response to bacteria such as *Mycobacterium tuberculosis* [6].

**Fig. 1:**
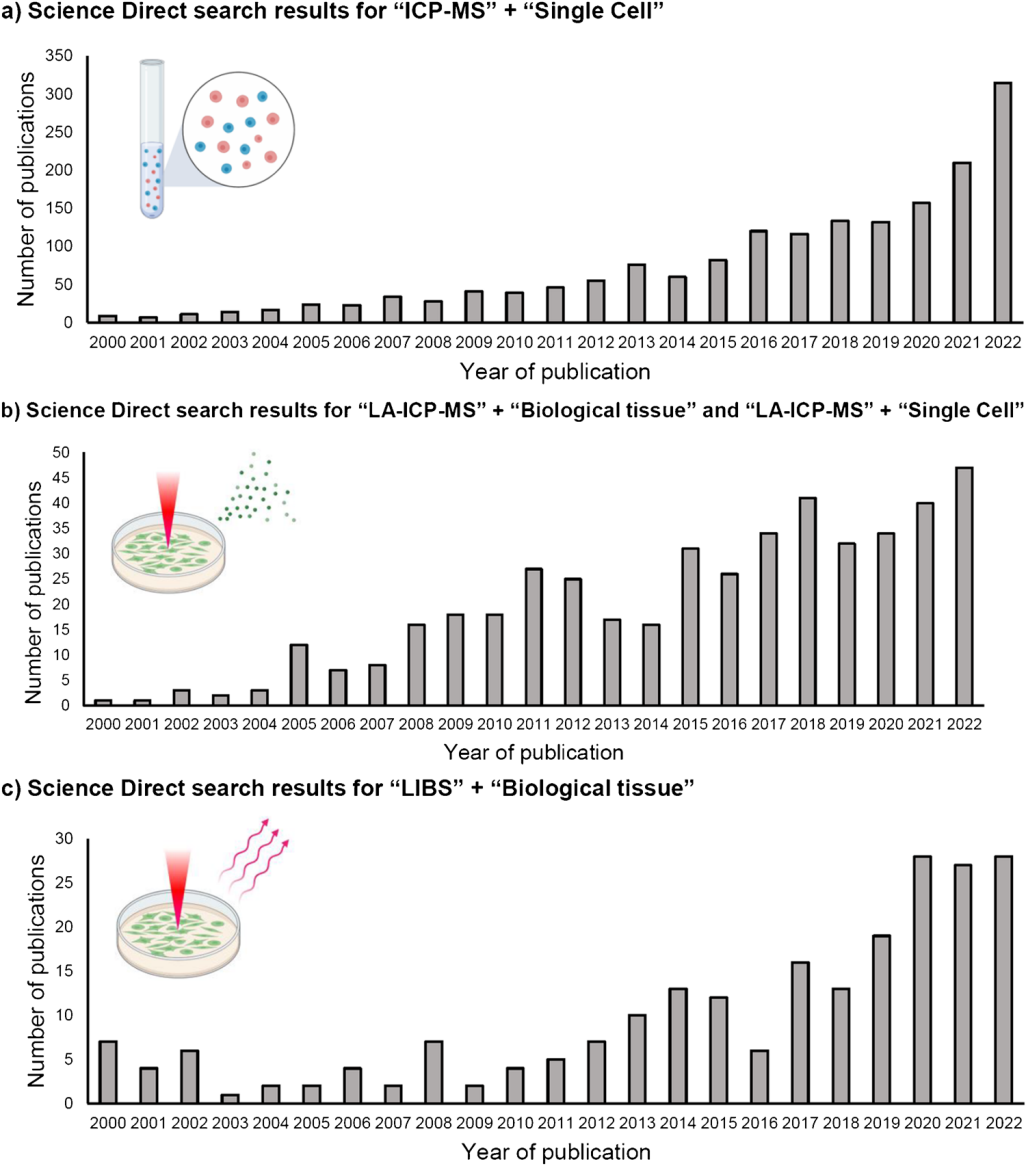
Number of research publications listed on Science Direct from 2001 to 2022, containing the words **a** “ICP-MS” and “Single Cell”, **b** “LA-ICP-MS” and “Biological tissue”/ “Single Cell” and **c** “LIBS” and “Biological tissue”

The study of elements in mammalian cells and tissues is critical to the understanding of important human diseases. Studying cellular responses to external stimuli and the impact of elemental distributions on both susceptibility to infection and drug efficacy will contribute to the development of novel treatments [1]. Of equal importance is the elucidation of the mode of action and cellular uptake of metal-containing drugs to develop more targeted therapeutic treatments [7]. The development of methods for the quantification of metallic elements is an important focus of bioanalytical chemical research (Fig. 1).

### Bulk analysis of biological samples

Until recently, quantitative elemental data was predominantly obtained from the analysis of bulk populations of cells yielding averaged data. This requires disruption of large numbers of cells followed by the analysis of the resulting bulk sample using a well-defined spectroscopic technique, most commonly solution ICP-MS (inductively coupled plasma-mass spectrometry) [8]. ICP-MS is commonly referred to as the “gold standard” for the elemental analysis of solutions, with high sensitivity, selectivity and linear dynamic range, providing quantitative multi-elemental capabilities [9].

There are, however, significant limitations when pairing a spectroscopic technique with bulk analysis. Firstly, the assumption is made that the population is normally distributed, and therefore, the average measurement is representative of the population. However, it has been shown that this is frequently not the case [10]. Secondly, sub-populations of cells can impact disease progression and treatment success, and this information on cellular heterogeneity is lost on bulk analysis. The integrity of each cell is destroyed during the extraction process, with the instrument providing an average concentration for the elements [11]. Methods to determine cellular heterogeneity are critical to fully understanding biological phenomena including disease progression, immunological bystander effects and antibiotic resistance.

### Atomic spectroscopy techniques for single-cell analysis

Single-cell analysis procedures must be able to detect analytes at extremely low concentrations (in the range of femtograms per cell [12]). To accurately quantify these elements at the single-cell and sub-cellular levels, high sensitivity and detection power are required. Time-resolved single particle-ICP-MS (SP-ICP-MS), otherwise known as single cell-ICP-MS (SC-ICP-MS), is a technique for the elemental analysis of cells in suspensions, in which short integration times are used to detect individual particles using conventional ICP-MS instrumentation [13]. Both solution ICP-MS and SP-ICP-MS allow for the quantitation of trace metals with parts per billion (ppb) levels of sensitivity; however, the critical difference is that SP-ICP-MS measures elements at a single-cell level rather than generating average values (“SP-ICP-MS”) [14]. Thereby, endogenous metals [15, 16] and exogenous metal-labelled drugs [17] within individual cells can be quantified, capturing cellular heterogeneity. Although the complex nature of cell culture media (often containing amino acids, lipids, proteins and inorganic salts [18]) requires some sample preparation, this is significantly less cumbersome than that required for bulk analysis, reducing the use of hazardous substances, which minimises environmental and economic costs [8].

**Fig. 2:**
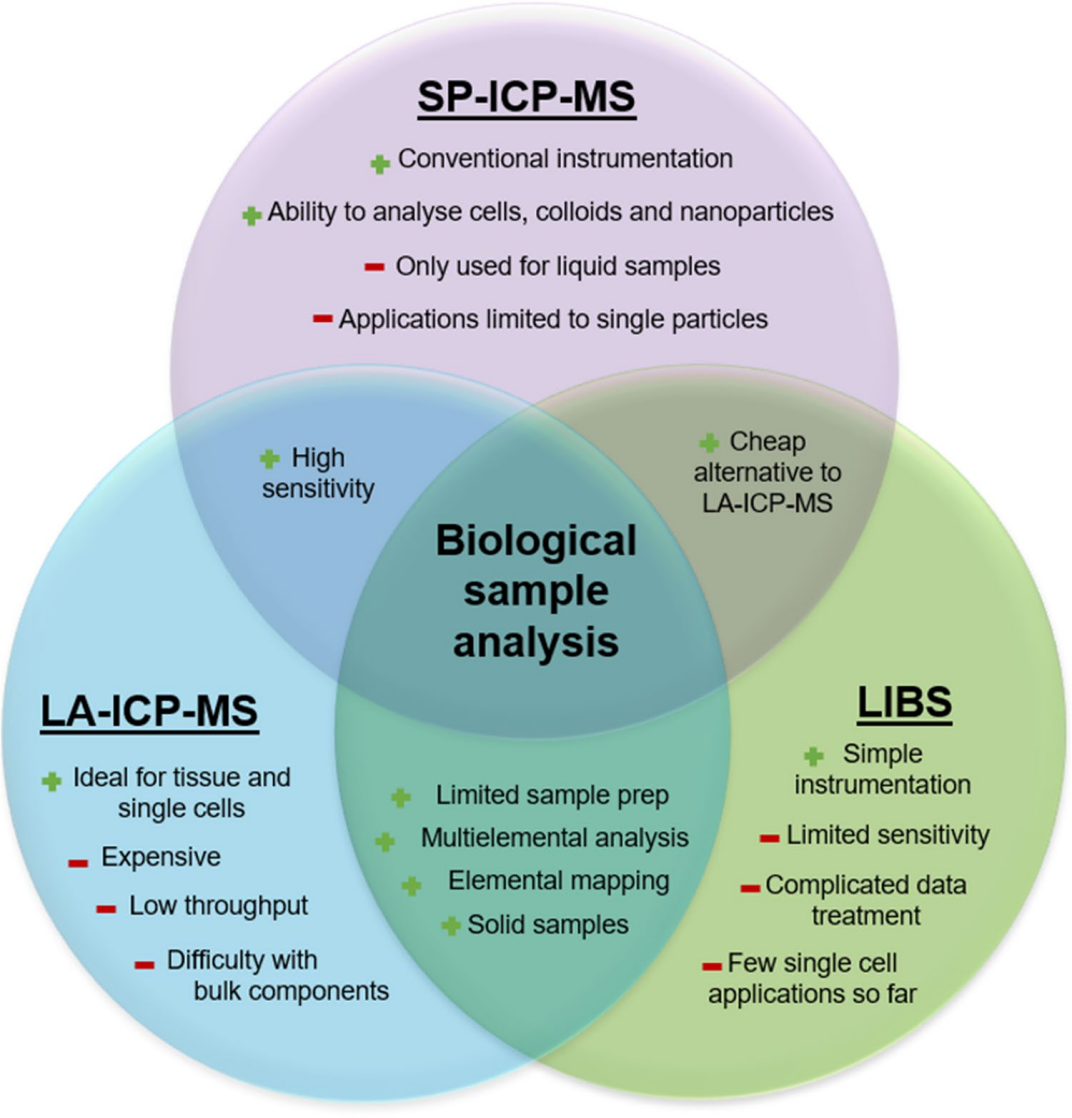
Comparison of main features of SP-ICP-MS, LIBS and LA-ICP-MS for biological sample analysis

Whilst for SP-ICP-MS the analysis is restricted to whole cells, the use of laser ablation (LA) for sample introduction provides spatial information with little sample preparation, paving the way for subcellular analysis [19, 20]. Spatially targeting and ablating specific cells may also allow bystander effects to be studied amongst cells in close vicinity [21]. Furthermore, the flexibility and simplicity of LA allows different detectors to be coupled with this sample introduction system, including atomic emission spectrometers (laser-induced breakdown spectroscopy, LIBS) [19] and the more sensitive mass spectrometers (LA-ICP-MS) [14]. LA-ICP-MS provides powerful detection required for both tissue and single-cell spatial analysis with ppb limits of detection, exceeding typical sensitivities associated with other direct analysis techniques such as particle-induced X-ray emission (PIXE) or X-ray fluorescence (XRF), albeit with inferior spatial resolution [22, 23]. LIBS, with reported limits of detection ranging from 1 to 100 parts per million (ppm), is not as sensitive as LA-ICP-MS [24], but it can analyse bulk biological components such as hydrogen, oxygen, nitrogen and carbon [25, 26], making LIBS a popular alternative. Due to the lower sensitivity along with complex data treatment, the use of LIBS instrumentation for single-cell studies has yet to be developed and explored in depth [9]. Increased analytical power can be achieved by combining SP-ICP-MS, LIBS and LA-ICP-MS (Fig. 2) techniques for biological samples [27, 28].

Atomic spectroscopy techniques are critically reviewed here, and future bioanalytical developments are discussed with an emphasis on their complementary nature for cellular analysis. This review will focus on the development of atomic spectroscopy technologies for the determination of endogenic elements, toxic metals/metalloids (resulting from environmental exposure) or metal-containing drugs that may be present in cells. The application and advances in mass cytometry, based on ICP-TOF (time-of-flight) instruments (CyTOF™), and imaging mass cytometry (LA-CyTOF-MS, SIMS) are not within the scope of this review. These technologies are based on the use of metal-containing antibodies to tag specific molecular components of the cells and do not allow for the detection of many intrinsic elements as the performance of the mass analyser has been optimised for tagged masses starting at 75 amu. CyTOF™ and LA-CyTOF-MS have been reviewed in detail elsewhere [29–33].

## SP-ICP-MS

**Fig. 3:**
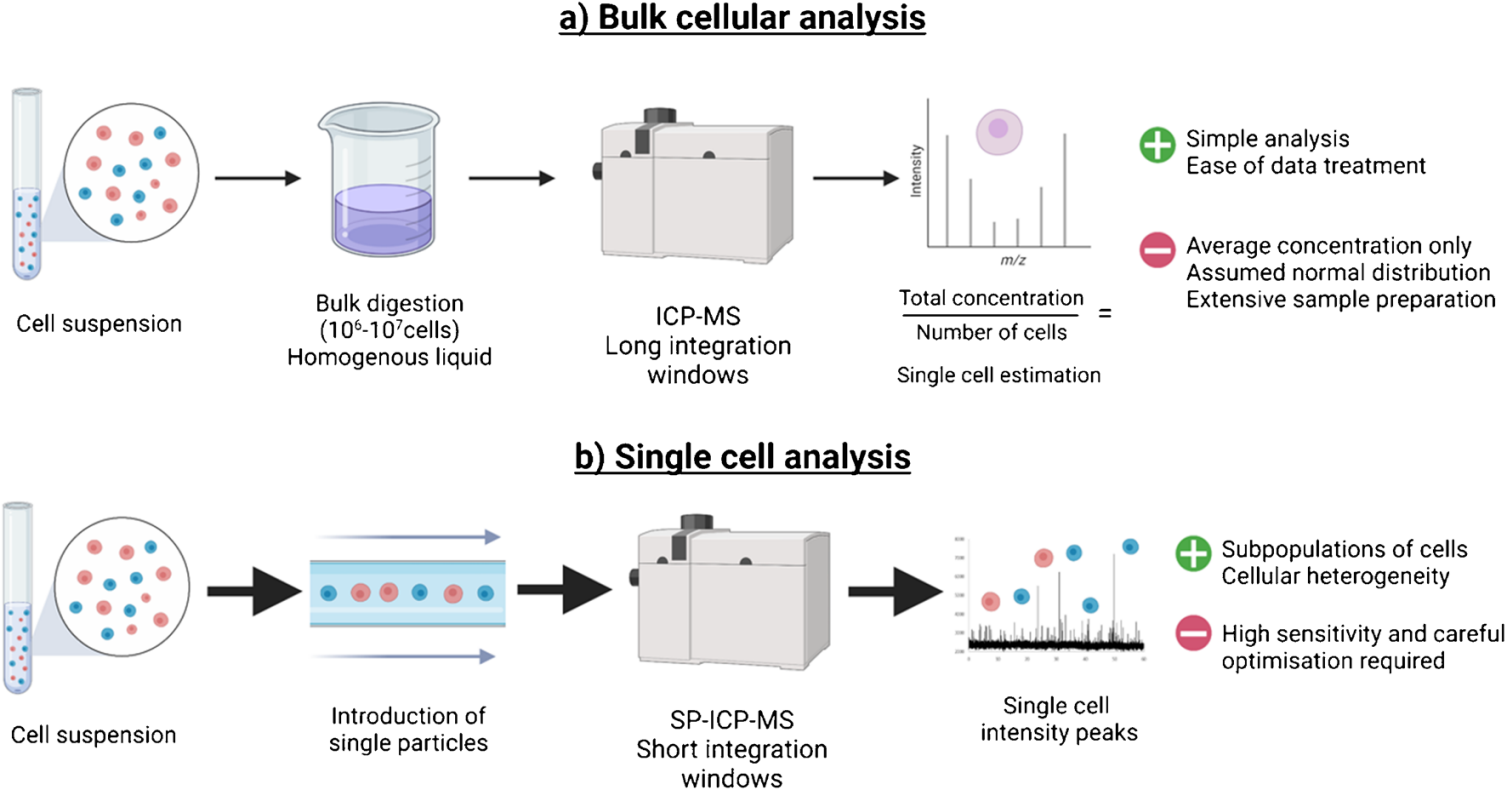
Comparison of main features of bulk solution ICP-MS and SP-ICP-MS for cellular analysis

Single particles (nanoparticles, colloids, prokaryotic and eukaryotic cells) can be analysed using the time-resolved analysis mode of conventional ICP-MS instrumentation. The duration of the integration time is the key difference between conventional solution ICP-MS and SP-ICP-MS; in bulk ICP-MS, the homogenous liquid sample is measured with relatively long integration time (seconds), whereas in SP-ICP-MS, the integration times are shorter (micro- or milliseconds). Particle suspensions are introduced continuously, and the use of short integration times allows thousands of transient count signals to be obtained in rapid succession. Most of these signals pertain to the sample matrix and constitute the background of the spectrum. When a single particle enters the ICP, an ion cloud is generated and the short dwell times enable the detection of this single-particle event as a result of the transient increase in the number of counts, which translates into the appearance of an intensity peak above the background (Fig. 3). When the system is optimised, each of these intensity peak signals correspond to individual particles, with the frequency of the spikes relating to the number of cells entering the plasma and the intensity of each particle event corresponding to the mass of analyte within the single particle [11]. For quantitative purposes in SP or SC-ICP-MS, it is critical to determine the *transport efficiency* of the samples, which is often expressed as a percentage, and it is defined as the ratio of the analyte entering the detector to the amount of analyte aspirated [13].

Quadrupole mass analysers are the most robust and commonly used for ICP-MS, and they have been successfully applied for the analysis of single cells (Table 1). However, these analysers only allow for the sequential detection of the ions; therefore, due to the high time resolution required for single-cell analysis, quadrupole technologies can only detect one element per single-cell event. The use of time-of-flight analysers (TOF) offers unparalleled time-resolution performance, as it allows the quasi-simultaneous detection of multiple elements in one single cell [34–37]. However, TOF instruments are not free of limitations in terms of sensitivity and their dynamic range [29]. Mass resolution is also critical for single-cell analysis, especially for the detection and quantification of some endogenous elements such as S, P, Fe and Se, which suffer acute spectral interferences. Although sector field (SF) mass analysers would provide the mass resolution required, their application to single-cell analysis has been limited so far [38]. The use of reaction/collision cell technology in ICP-MS/MS instruments, also known as ICP-QQQ, has been proven successful for the assessment of the cellular bioavailability of arsenite [39], and also of endogenous elements (including S and P) and Pt-chemotherapeutic drugs using O_2_ in the reaction cell for the monitoring of PO^+^ and SO^+^ [40].

**Table 1:** Examples of research publications in SP-ICP-MS analysis, detailing elements, sample type, instrument, quantitative/qualitative nature, calibration approach and reported approximate transport efficiency

Application	Elements	Sample	ICP-MS*	Quantitative/Qualitative	Calibration	Transport efficiency (%)	Reference
Endogenous elemental analysis	Cu, Mg, Mn	Algae	Agilent 7500a (Q)	Quantitative	Dissolved standard solution, MgO particles	0.54	[43]
	Ca, Mg	Bacteria	Thermo Finnigan Element 1 (M Sec)	Qualitative	N/A	–	[38]
	Cu, K, Mg, Mn, Zn	Yeast cells	Agilent 7900 (Q)	Quantitative	Dissolved standard solution	5.1	[12]
	Ca, Cu, Fe, Mg, Mn, P, Zn	Yeast cells	Agilent 7700x (Q)	Semi-quantitative	ICP-OES digestion	75	[15]
	Al, C, Ca, Cr, Fe, K, Mg, Mn, P, S, Zn	Yeast cells, cyanobacterium, algae	Agilent 7500a (Q)	Qualitative	N/A	86–100	[16]
	Zn	HepG2 cells	Thermo X Series II (Q)	Quantitative	ZnO nanoparticles	3.0	[52]
	Cu	Red blood cells	PerkinElmer NexION 300X (Q)	Quantitative	Dissolved standard solution	100	[51]
	Fe, Mg, P, S, Zn	Yeast cells, algae, red blood cells	Agilent 8900 (QQQ)	Quantitative	Dissolved standard solution	8–13	[48]
	Cu, K, Mg, Mn, P, Zn	Yeast cells	Thermo iCAP Q (Q)	Quantitative	Dissolved standard solution	0.3	[46]
	Mg	Bacteria	Agilent 7500a (Q)	Quantitative	MgO nanoparticles	–	[56]
	Fe	A2780 cells	Agilent 7700 (Q)	Quantitative	Dissolved standard solution	25	[49]
	Mg	Algae	PerkinElmer NexION 300D (Q)	Quantitative	Dissolved standard solution	46–64	[55]
	Mg	Algae	Agilent 7500a (Q)	Semi-quantitative	Dissolved standard solution	0.6	[44]
	Cu, Fe, Mg, Mn, P, Zn	Algae	TOFWerk icpTOF S2 (TOF)	Qualitative	N/A	–	[34]
	P	Yeast cells	TOFWerk icpTOF 2R (TOF)	Qualitative	N/A	–	[35]
	Ca, Fe, Mg, P	Yeast cells, algae	TOFWerk icpTOF (TOF)	Quantitative	Dissolved standard solution	–	[36]
	Fe, Mg, P, Si	Diatom species	TOFWerk, icpTOF R & icpTOF 2R (TOF)	Qualitative	N/A	1	[37]
	Cu, Fe, P, S, Zn	Raji, Jurkat and Y79 cells	Agilent 8900 (QQQ)	Quantitative	Dissolved standard solution	60	[40]
	C	Algae	Agilent 8900 (QQQ)	Quantitative	Polystyrene-based microplastics	2–6	[41]
Cellular uptake of drugs, nanoparticles and metals	Cr	Algae	Agilent 7500a (Q)	Quantitative	Dissolved standard solution, MgO particles	0.54	[43]
	U	Bacteria	Thermo Finnigan Element 1 (M Sec)	Qualitative	N/A	–	[38]
	Cu	Yeast cells	Agilent 7900 (Q)	Quantitative	Dissolved standard solution	5.1	[12]
	Zn	HepG2 cells	Thermo X Series II (Q)	Quantitative	ZnO nanoparticles	3.0	[52]
	P	Yeast Cells	Thermo iCAP Q (Q)	Quantitative	Dissolved standard solution	0.3	[46]
	Bi	Bacteria	Agilent 7500a (Q)	Quantitative	MgO nanoparticles	–	[56]
	Pt, Tb	A2780 cells	Agilent 7700 (Q)	Quantitative	Dissolved standard solution	25	[49]
	Gd, Pt	Hela, 16HBE cells	Thermo X7 (Q)	Quantitative	Dissolved standard solution	–	[17]
	Cu	Algae	PerkinElmer NexION 300D (Q)	Quantitative	Dissolved standard solution	46–64	[55]
	Au	Algae	PerkinElmer NexION 300D (Q)	Quantitative	Dissolved standard solution, Au nanoparticles polystyrene beads	30–35	[50]
	Ba	Algae	TOFWerk icpTOF S2 (TOF)	Qualitative	N/A	–	[34]
	Pb	Yeast cells	TOFWerk icpTOF 2R (TOF)	Qualitative	N/A	–	[35]
	Ru	Yeast cells, algae	TOFWerk icpTOF (TOF)	Quantitative	Dissolved standard solution	–	[36]
	Zn	Diatom species	TOFWerk, icpTOF R & icpTOF 2R (TOF)	Qualitative	N/A	1	[37]
	Pt	Raji, Jurkat and Y79 cells	Agilent 8900 (QQQ)	Quantitative	Dissolved standard solution	60	[40]
Speciation	Cr (III), Cr (VI)	HeLa, MCF-7 cells	Agilent 7500a (Q)	Quantitative	Dissolved standard solution	1	[45]
	Cr (VI)	Algae	Agilent 7500a (Q)	Semi-quantitative	Dissolved standard solution	0.6	[44]
	As (V)	Algae	PerkinElmer NexION 300 X (Q)	Quantitative	Dissolved standard solution	9.9	[47]
	As (III)	A549 cells	Agilent 8800 (QQQ)	Quantitative	Dissolved standard solution	0.5	[39]

^*^Q single quadrupole, *QQQ* triple quadrupole, *M Sec* magnetic sector, *TOF* time of flight

González de Vega et al. [41] investigated the different acquisition modes and collision/reaction gases to optimise the detection of ^12^C or ^13^C to characterise the uptake of microplastics in unicellular algae. Despite their success in removing spectral interferences, QQQ mass analysers operate sequentially as per single-quadrupole instruments, and Liu et al. [40] pointed out that the use of the collision/reaction cell may result in a change in the duration of the “single event” through the mass analyser; therefore, further research is needed to adjust the dwell times for those “anomalously longer” signals.

### Initial proposals and development

The application of ICP-based spectroscopy for single-cell analysis was first identified by Nomizu et al. [42] who proposed a technique for the analysis of calcium in individual mouse fibroblast cells using inductively coupled plasma optical emission spectroscopy (ICP-OES). Sample introduction efficiency (< 0.1%) required improvement, and a lack of sensitivity restricted the application of the technique to other elements. The authors identified ICP-MS as a potential solution to overcome these limitations, but this was not explored until over 10 years later [38]. This seminal study provided the catalyst for research into the analysis of single cells using ICP-MS.

**Fig. 4:**
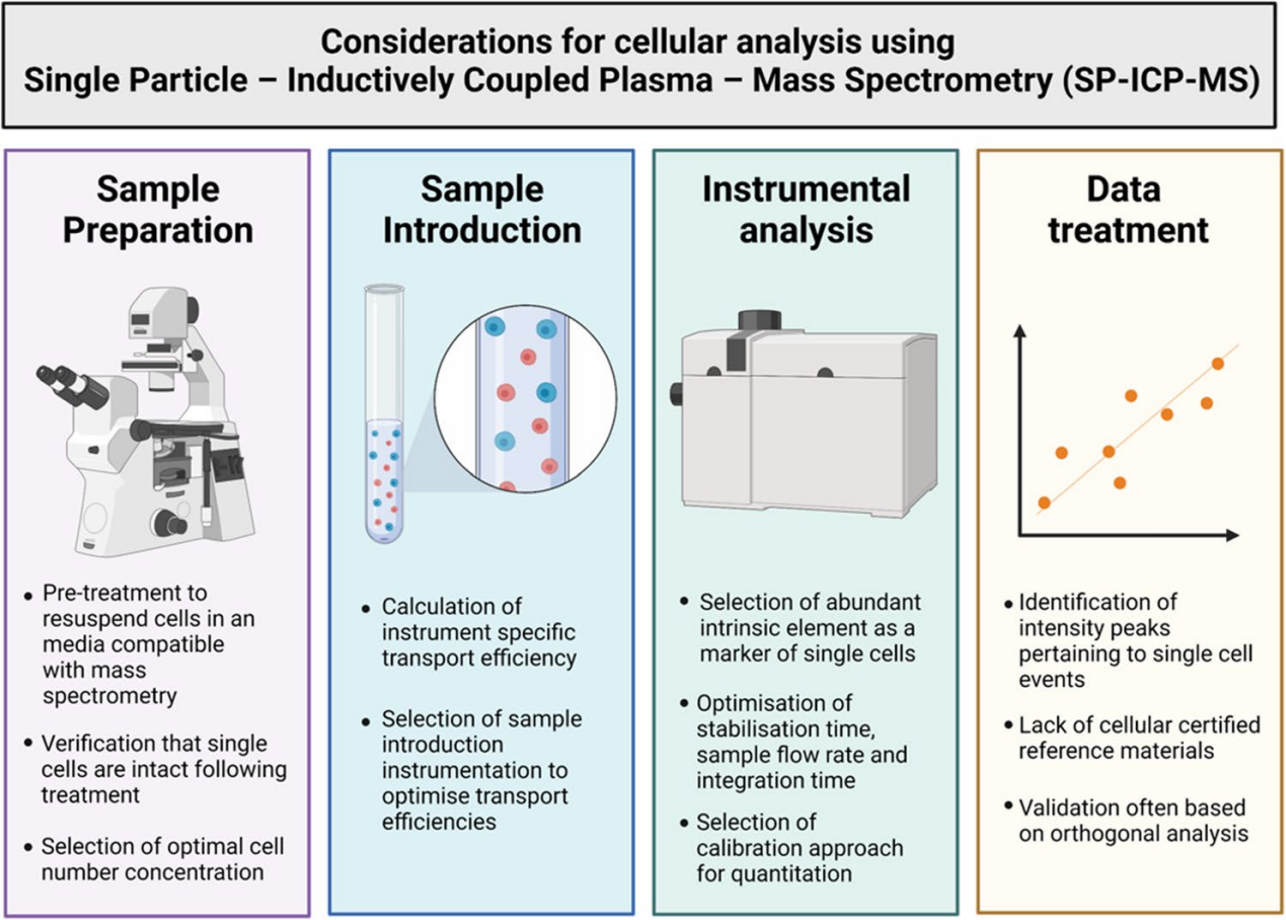
Considerations surrounding sample preparation, sample introduction, instrumental analysis and data treatment for cellular analysis using SP-ICP-MS

SP-ICP-MS was first developed for single-cell analysis by Li et al. [38], who applied SP-ICP-MS paired with perfusion chromatography to measure uranium in *Bacillus subtilis*. This study focused on ensuring that the cells were intact upon introduction. However, the significant increase in intensity of signals when measuring cell suspensions of different concentrations suggested that the cells were not introduced into the instrument one at a time. For accuracy, it is critical that the cells are both intact and introduced as single particles. An inorganic standard solution was used for calibration with the assumption that this had the same transport, atomisation and ionisation efficiency to the cells. As highlighted in these early publications [38, 42], it is critical for accurate measurements that there is efficient introduction of the sample into the instrument and that calibration techniques are developed so that differences in ionisation and transport efficiencies between the standards and the test samples are accounted for (Fig. 4).

### Instrumental design for improvement of transport efficiency

The accuracy of SP-ICP-MS measurements is dependent on the ability of the instrument to uptake and introduce single particles. The aerosolization of a cell suspension using a pneumatic nebuliser is a random process, and the formation of a single ion cloud produced by multiple particles is a possibility that must be minimised. Moreover, conventional introduction systems suffer from low transport efficiencies (“Calibration and validation for quantitative analysis”). Transport efficiencies have been reported to be as low as 0.5% [43], 0.6% [44] and 1.0% [45] using a standard V-groove nebuliser for aerosol generation, and even lower when using a cyclonic spray chamber (~ 0.3%) [46]. Therefore, the development of novel devices to reduce introduction uncertainty and improve transport efficiencies is at the forefront of SP-ICP-MS research.

A high-efficiency introduction system consisting of a perfluoroalkoxy nebuliser and Asperon spray chamber was used to obtained transport efficiencies of 9.9% [47]. The authors used Poisson statistics to predict the likelihood of multiple-cell events; however, this approach to evaluate the uncertainty of single-cell introduction does not account for cell aggregation but rather relies on a study of the duration of cell events and the relationship with the sample flow rates and cell number concentration [47]. Tanaka et al. [48] considered it prohibitive for many ICP-MS users to access expensive or enhanced devices and found a higher transport efficiency (~ 10%) could be obtained for yeast, green alga and red blood cells using a standard concentric nebuliser, presenting a significant improvement to the values found previously (~ 1%).Further improvements were made with the EnyaMist nebuliser and a total consumption spray chamber used to quantify cisplatin uptake in single cells [49], achieving transport efficiencies as high as 25%. Transport efficiencies of 30–35% have been reported when using an Asperon spray chamber and a high-efficiency concentric glass nebuliser. This setup was designed to increase the transport efficiency by reducing cell damage and collision with the chamber walls and showed a significant improvement compared to a cyclonic spray chamber [50]. The SP-ICP-MS setup for the elemental analysis of yeast cells described by Groombridge et al. [15] incorporated a modified high-performance concentric nebuliser and a low-volume on-axis spray chamber, achieving transport efficiencies of 75%. Miyashita et al. [16] developed a novel high-efficiency cell introduction system, building on previous work by reducing cell loss to the spray chamber walls and modifying the system to allow different cell sizes to be introduced with high efficiencies (86 to 100% for yeast, cyanobacterium, red and green algal cells). Recently, a single-cell introduction system with 100% efficiency was developed [51] consisting of a flow cell, visual contrast calibration device and customised nebuliser and spray chamber used for the quantitation of copper in single human red blood cells.

Although improvements in the design of the nebuliser and spray chamber may result in enhancement of the transport efficiency, they do not remove altogether the risk of multiple-cell events in the plasma. Thus, the design of devices that allow for the manipulation of cell suspensions based on microfluidic chip technology has been proposed to improve accuracy by ensuring the controlled introduction of single cells. An example of such developments was presented for the measurement of zinc in HepG2 cells [52]. The aqueous suspension was separated into single-cell-containing droplets using a flow-focusing geometric design, optimising key parameters such as the organic phase, geometric structure, chip-to-instrument interface and flow rates. The detection efficiency was still low at 3%, but there was a high throughput of cells at 3–6 × 10^6^ droplets per minute, and the system was highlighted as being easy to develop and operate. Wei et al. [53] developed a single-cell pipette microfluidic chip composed of a Z-shaped channel and a horizontal linked microchannel that enable the capture of single cells into nanolitre droplets, without compromising the integrity of the cells. Chen et al*.* [54] developed a droplet microchip that used oil/gas phase to encapsulate single cells in droplets. This was then coupled with a negative magnetophoresis-focussing microchip to remove the oil phase prior to the entry of the cell into the plasma, achieving a throughput of dozens of cells per minute [54].

### Method development

Optimisation of instrumental parameters and sample preparation procedures is critical for accurate analysis using SP-ICP-MS. The analyst should aim to reduce the probability of registering signals produced by the overlap of multiple cells (overestimation of the particle signal) or of one cell being split into multiple signals (underestimation of the particle signal). Method development predominantly focuses on cell number concentration, integration time and sample flow rate [17, 55].

Accurate SP-ICP-MS analysis begins by selecting the optimum cell number concentration. This needs to be sufficiently low to ensure single cells are entering the plasma, but high enough to obtain a statistically significant number of peaks. Triplicate measurements of cells counted using light microscopy and haemocytometers or cell counters are often used for quantifying cell number in test suspensions [15–17, 43]; however, flow cytometry has also been used [38, 49]. An intrinsic element within the cells is often used as a marker to assess the impact of parameters on the intensity and number of analyte peaks. Magnesium is a common choice due to its abundance in living cells and high ionisation efficiency [43, 44, 56]. Other elements such as phosphorus [15] and copper [51] have also been used. Cellular concentrations from ~ 8 × 10^4^ cells/mL to 4 × 10^6^ cells/mL have been shown to yield optimum results with ICP-MS [15–17, 47, 57]. This large variation reflects natural differences in cell sizes and sample introduction instrumentation. When the optimal cell density is selected, dilution of the suspension should lead to a linear decrease in the number of peaks obtained; however, the range of count intensities of the peaks pertaining to single particles should remain relatively consistent [42]. Groombridge et al. [15] found the correlation between the cell density and the number of intensity peaks for ^31^P^+^ to be linear, demonstrating the low probability of the recorded events being caused by more than one cell. Similarly, at higher concentrations, the peak intensities will eventually increase as multiple cells begin entering the plasma per integration time.

Limited attention has been paid to sample treatment prior to introduction into the ICP. This is particularly important as a high signal-to-noise ratio is required to distinguish single-cell atomisation events from the baseline background signal. Culture media required to support cellular growth presents high total dissolved solids (TDS), which originates a high background signal, effectively hindering the accurate distinction of single-cell events. The direct introduction of cells in this complex media also increases the potential for nebuliser blockages, impacting plasma stability, accuracy and precision [58]. In some cases, cells (*Saccharomyces cerevisiae* [15]) can be directly resuspended in water; however this approach is not possible with most mammalian cells and pre-treatment is often required. Fixation aims to preserve the chemical and cellular composition in a life-like state, preventing the degradation of cellular components and structures and rendering them sterile [59]. For the study of infectious processes, sterilisation methods are essential. Methanol [57] and paraformaldehyde fixation [60] have been used so that non-adherent cells can be resuspended in water without osmotic lysis. Aldehyde fixative agents have been found to leach metals from biological tissue [61, 62], highlighting the need for an in-depth assessment of the effect of different fixation agents on elemental concentrations in single cells to ensure accurate measurements. Due to the time-consuming fixation procedures and the risks of cell rupture or leaching, other researchers have opted for the coupling of separative techniques such as liquid chromatography (LC) [37] or asymmetrical flow field-flow fractionation (AF4) [35], to achieve the cleaning of the cellular suspensions and reducing the ionic background caused by the culture media or cellular debris. Von der Au et al. [37] developed a fully automated cleaning and introduction system to characterise the concentration of Mg, P, Si and Fe in diatomaceous algae to evaluate ecological stress. This approach was based on the use of a flow injection HPLC system set with two valves to allow the automatic washing of the cell culture directly coupled with an ICP-TOF–MS [37]. Cronakis et al*.* [35] coupled the same mass analyser to a AF4 system using sodium dodecyl sulphate (SDS) and NaN_3_ as the carrier phase, for the determination of metals in baker’s yeast and confirming by flow cytometry and scanning microscopy that the separation procedure did not affect the cellular integrity. These techniques are very attractive due to their high degree of automatization and reduction of background levels; however, these approaches were developed using yeast cells and diatoms and will require further development for their application to a wider range of eukaryotic and prokaryotic cells ([63, 64] for details on the cellular structure of yeast and diatoms).

Integration or dwell time is also an important parameter in SP-ICP-MS. Whilst solution ICP-MS uses long integration times in which multiple readings measure the total metal concentration of a sample, SP-ICP-MS uses short integration times to record individual signals [13]. However, excessively short integration times can lead to incomplete measurements and an increase in background [45]. Sample and gas flow rates are also critical for accurate quantification using SP-ICP-MS [38, 43]. In terms of data treatment, an algorithm for separating single-particle events from the background signal based on three standard deviations of the complete data set [13] has been successfully applied [51, 52]. Note that the use of short integration times of 50 µs and low concentrations of analyte can cause a deviation from the normal distribution of the background signal and require Poisson statistics to determine the limit of detection [47].

### Calibration and validation for quantitative analysis

Reports of calibration and validation procedures have been limited due to a lack of suitable cellular standards. Existing approaches share the postulation that ions from a dissolved standard solution and those from a nanoparticle or cell will behave in a comparable fashion. A study investigating the elemental concentrations of single *Chlorella vulgaris* [43] using aqueous calibration standards of magnesium highlighted a difference of 3.11 × 10^8^ atoms of magnesium per cell between SP-ICP-MS using aqueous calibration standards and conventional solution ICP-MS. This variation is likely due to differences in transport efficiencies and diffusion loss—again emphasising that data analysis methods which take these into account must be applied. Accuracy was improved when repeating the calibration using a MgO particle suspension instead of aqueous standards. SP-ICP-MS was able to determine cell numbers within an unknown suspension by using a calibration curve of density of cells against the Mg spikes [43]. This MgO nanoparticle calibration method also validated the introduction of single *Helicobacter pylori*, the causative agent of gastritis [56]. Here, the values obtained using SP-ICP-MS and solution ICP-MS were in concordance, confirming that single cells had been successfully introduced into the instrument.

Quantitative analysis of SP-ICP-MS must account for differences in transport efficiencies and atomisation properties between test samples and calibration standards. Transport efficiency, often expressed as a percentage, is defined as the ratio of the analyte entering the detector to the amount of analyte aspirated [13]. Li et al. [38] emphasised that accurate quantification required differences in transport efficiencies between samples and standards to be included in any calculations. Pace et al. [13] compared waste and sample-uptake volumes, but this led to overestimation of transport efficiency. A well-characterised reference suspension of a known particle size was used as an alternative method, whereas the particle frequency method used a reference suspension of a known particle number concentration to determine transport efficiency. This marked a turning point in SP-ICP-MS research as the first attempt at defining standard calibration techniques and data analysis [13]. The accuracy of these approaches relies heavily on the similarity of particle size between the biological samples and transport efficiency reference material [47, 48]. Significant differences between transport efficiencies of ~ 5-µm cells (8–13%) and 200-nm silica nanoparticles (55%) were reported, highlighting that a suspension of comparable size to the real sample must be used for optimum accuracy [48]. Alternative approaches used gold reference nanoparticles [49, 50] and analyte-doped polystyrene beads with a diameter and population density matching that of the cell suspension to determine transport efficiencies [50]. Miyashita et al. [16] also reported cell introduction efficiencies decreased with increasing cell size, emphasising the importance of comparable sizing between test and reference samples.

In some reported cases, significant differences between the arsenic uptake calculated by SP-ICP-MS (1.5–1.8 fg of As) and conventional ICP-MS (3.0–8.0 fg of As) were found when bulk ICP-MS was used to validate results [47]. A 20% underestimation of measurements using SP-ICP-MS was reported due to transport efficiency variations between different-sized cells, incomplete ionisation and inconsistent single-cell introduction [46]. Whilst solution ICP-MS is a highly sensitive validation approach and therefore commonly used [50], this technique only shows an average analyte concentration. Discrepancies can be due to differences in transport efficiency between standard and sample, changes in suspension conditions and inaccurate cell counting, reflecting issues in using a bulk measurement technique to validate a single cell measurement. As matrix-matched certified reference materials are scarce for biological samples, SP-ICP-MS must be validated using other methods. In this respect the use of LA techniques alongside SP-ICP-MS is very promising [17]. An alternative approach used flow cytometry coupled with ultra-fast LC–MS and SP-ICP-MS to measure *Microcystis aeruginosa* and the effectiveness of copper-based algaecides [55].

### Expansion of applications

SP-ICP-MS is a critical technique to improve our understanding of single cell behaviour (Table 1). However, this has barely scratched the surface of the potential biological applications of SP-ICP-MS. Several studies [15, 16] have focused on method development with “model organisms”, such as the yeast *Saccharomyces cerevisiae*, which is a model for eukaryotic cells. This is a well-defined system with advantages for biological studies including ease of manipulation, rapid growth and similarities with the human genome [65]. However, from an analytical perspective, it will be important to consider a wider scope of cell sizes and types, as SP-ICP-MS can present issues relating to poor sample introduction that are heavily influenced by the sample matrix and cell type.

Another direction of research which needs readdressing is validation, as the use of standard solutions for calibration is questionable. The quantification strategies described assume that all the cells in the population have the same dimensions/cellular volumes, resulting in a biased description the heterogeneity of the metal concentration in the cell population. To tackle this issue, González de la Vega et al. [41] proposed the quantification of carbon to calibrate the physical dimension of the cell using either ^12^C or ^13^C signal detected by SC-ICP-MS/MS. Although this approach proved successful for the evaluation of the size distribution of seawater algae Symbiodiniaceae, the authors did not show any data for the simultaneous quantification of the C and other metals in the same cells with the QQQ instrument. Qin et al. [36] reported the simultaneous analysis of Ca and Fe in *Saccharomyces cerevisiae* using ICP-TOF–MS and proposed the use of ruthenium red in staining to increase the visibility of the cells and provide a normalisation parameter that accounts for the variability in cell volumes. The authors concluded that the use of Ru provided a better correlation with cell volume than elements commonly used such as Mg and P. Many studies rely on solution ICP-MS for validation [17, 50], which may confound the results. In future, attempts should be made to compare and couple SP-ICP-MS to other single-cell techniques (such as LA-ICP-MS or PIXE) for full quantitative analysis.

## LIBS

LIBS presents advantages for bioanalytical applications when compared to techniques that require substantial sample preparation (solution ICP-MS [8]) or expensive and highly specialised operational facilities (PIXE [66]). However, as the ablation process removes a small amount of mass (~ 10 µg) [67] from the sample surface, damage to the sample paired with differences in ablation efficiency and disparity in ablation depth can originate average to low precision of replicate measurements (≤ 10% relative standard deviation) [68, 69]. Therefore, careful optimisation of laser parameters (laser wavelength, number of shots, pulse energy, warm-up shots and delay time) is required for rapid multi-elemental detection of trace and major elements. This is a simple instrument which works at room temperature/atmosphere requiring limited sample preparation [9], and can be coupled to other techniques from the simple (e.g. optical microscopy) to the more sensitive (e.g. LA-ICP-MS) [25]. Therefore, LIBS has been applied to biological samples including tissues [70] and bacteria [71, 72]. However, this technique has relatively low sensitivity (low ppm range [24]) and is still in its infancy for sub-tissue applications [73]. Although accuracy of the technique is limited by the lack of certified reference materials and complex calibration and data analysis methods [22], coupling LIBS to other techniques can overcome some of these limitations and is predicted to become an important area of future research.

### Applications in bioanalytical chemistry

Early studies demonstrated that LIBS is a sensitive tool for elemental analysis of biological samples in the solid state. Samek et al. [74] compared the concentrations of calcium and phosphorous in healthy and decayed teeth. LIBS has also been successfully applied for in vivo monitoring of the laser drilling process involved in dental treatments [75]. LIBS proved a simple, effective and cheap method for evaluating the ability of protective creams to limit zinc absorption into human skin [76]. LIBS was used for the mineral analysis of hair tissue [77], highlighted for presenting advantages over conventional ICP-MS in terms of cost, sample treatment and elemental mapping capabilities. Others have attempted to differentiate healthy and cancerous tissue by LIBS measurements of Ca, Cu, Na and K [78]. Despite developments (Table 2), the sensitivity of LIBS remains the major limitation. Biological advances include the validation of a rapid accurate method to identify lymphoma in blood samples [79], a calibration-free approach to quantify gallbladder stones [80] and elemental imaging of paraffin-embedded skin samples [81]. LIBS applications have focused on elemental analysis and mapping of tissues rather than single-cell analysis, which requires increased sensitivity (femtogram per cell range) [82]. The potential to extend the applications of LIBS into the single cell requires further investigation. Nanoscale-LIBS imaging promises improved sensitivity [73], utilising both a femtosecond laser for sampling and a nanosecond laser for emission enhancement. This nanoscale-resolution LIBS imaging has been applied to the analysis of indium phosphide nanoparticles in single cells, with limits of detection of the technique reported to be in the femtogram range (“Potential for single-cell analysis”).

**Table 2:** Examples of research publications in LIBS analysis, detailing elements, sample type, laser used, quantitative/qualitative nature and calibration approach

Application	Elements	Sample	Laser (nm)	Quantitative/ qualitative	Calibration	Reference
Endogenous elemental analysis in tissue	C, Ca, H, K, Mg, N, Na, O	Porcine fat and nerve tissue	532	Qualitative	N/A	[70]
	Zn	Human skin	1064	Quantitative	PMMA + ZnCl_2_/ethanol droplets	[68]
	Ca, P	Human teeth, bones	–	Qualitative	N/A	[74]
	Ba, C, Ca, K, Li, Mg, Na, P, Sr, Zn	Human teeth	1064	Qualitative	N/A	[75]
	Ca, K, Mg, Na	Human hair	1064	Quantitative	Calibration-free approach	[77]
	Al, Ca, Cu, Fe, K, Mg, Na	Dog liver	532	Qualitative	N/A	[78]
	Ca, Fe, K, Mg, Na	Whole blood	1064	Qualitative	N/A	[79]
	C, Ca, Cu, Fe, H, K, Mg, Mn, N, Na, O, P, Sr, Zn	Gallstone	532	Quantitative	Calibration-free approach	[80]
	Ca, Fe, K, Mg, Na, P	Human skin	1064	Qualitative	N/A	[81]
	C, Ca, Fe, H, K, Mg, N, Na, O	Porcine nerve, gland tissue	532	Qualitative	N/A	[84]
	Al, Pb, Sr	Human teeth, bones	1064	Quantitative	CaCO_3_-based pellets	[89]
	Cu, Mg, Sr, Zn	Human kidney stones	532	Quantitative	Calcium oxalate monohydrated–based pellets	[88]
	Ca, Co, Mg, Mn, Ni, Sr, V	Human teeth	266	Quantitative	Hydroxyapatite-based pellets	[86]
	C, Ca, I, K Li, Mg, Na	Rat thyroid, salivary, mammary glands	1064	Semiquantitative	Lithium-immersed tissue	[85]
	Ca, Na, Zn	Murine kidney tissue	532	Qualitative	N/A	[83]
	Ca, Cu, Fe, Mg, Na, P	Murine kidney tissue	266	Quantitative	Epoxy resin	[90]
	Ca, Cu, Fe, Mg, Na, P	Rodent kidney, tumour tissue	1064	Quantitative	Dried nanoparticle aqueous droplets	[87]
	Ca, Na	Murine kidney tissue	1064	Quantitative	ICP-OES digestion	[91]
Drug and nanoparticle cellular uptake in tissue	Zn	Human skin	1064	Qualitative	N/A	[76]
	Gd, Si	Murine kidney tissue	266	Quantitative	Epoxy resin	[90]
	Gd, Si	Rodent kidney, tumour tissue	1064	Quantitative	Dried nanoparticle aqueous droplets	[87]
	Gd	Murine kidney tissue	1064	Quantitative	ICP-OES digestion	[91]
Microbe identification	Al, Ca, Cr, Fe,, Na, Si	Pollen	1064	Qualitative	N/A	[93]
	C, Ca, K, Mg, Na, P	Mould and yeast	–	Qualitative	N/A	[71]
	Ca, K, Na	Fungal spores, bacteria	532	Quantitative	NaCl, CaNO_3_ and KCl particles	[94]
Cellular applications	K, Na	Red blood cells	193	Qualitative	N/A	[95]
	C, Ca, Cl, H, K, Mg, Na, O, P	HaCaT cell pellet	532	Qualitative	N/A	[96]
	In	Mouse mononuclear macrophage leukaemia cells	515, 266	Qualitative	N/A	[73]

### Calibration approaches for biological sample analysis

LIBS has successfully been used to analyse both hard and soft tissues; however, many of these studies only offer qualitative or semi-quantitative measurements [83–85]. A validated quantitative method using matrix-matched standards paired with an internal standard to correct for instrumental drifts, differences in ablation efficiencies and plasma conditions is not always possible for biological samples. Alternative calibration and validation approaches that do not rely on commercially available reference materials are therefore the focus of several studies [86–88]. Elemental analysis of ex vivo tissue removed by laser surgery used ratios of different element emission lines for relative quantification [70]. This work was extended to develop a feedback system to prevent nerve damage during laser surgery [84]. These semi-quantitative LIBS approaches are commonly used with biological samples where reference materials are not available.

Several approaches have been used to generate calibration standards that match the matrix composition of biological samples, for example, using ethanolic ZnCl_2_ solutions at different concentrations to create a calibration curve based on polymethyl methacrylate (PMMA) polymer-coated glass slides for skin analysis [68]. CaCO_3_ spiked with phosphorous compounds has been used to produce pressed pellets to mimic bone; however, attempts to reduce the brittleness of the pellets to match the biological samples were unsuccessful [89]. Calcium oxalate monohydrate mixed with standard solutions correlated well with the measurements obtained by conventional ICP-MS [88]. Matrix-matched hydroxyapatite standards to mimic teeth were used to analyse archaeological samples [86]. A variation of this technique was used for monitoring lithium treatment in a rat model [85]. Tissues from untreated control animals were immersed in various concentrations of lithium, and a calibration curve was generated from the measurements of these samples with the assumption that the signal for these controls correlated with the concentration of lithium in solution. However, this did not take into consideration differences in adsorption of the Li to the tissues.

Sancey et al. [87] used nanoparticle-based aqueous standards for calibration in gadolinium nanoparticle mapping of tissues. Improved ablation control and reduced matrix effects were achieved by using epoxy resin [90]. More recently, the use of paraffin embedding was explored by Moncayo et al. [81] for the first time, who used LIBS for the elemental imaging of healthy and malignant human skin tissues, allowing for a direct qualitative comparison between elemental images.

Alternative approaches without the use of external calibrators rely on independent validation using another technique. Gd-containing nanoparticles were imaged using LIBS and independently analysed with ICP-OES to determine the total mass of elements in each section of murine kidney [91], assuming a homogenous distribution of the elements. A calibration curve was produced by comparing the obtained mass to the LIBS intensity within different slices. This approach requires sufficiently large-sized tissue samples and is totally destructive. Spiking samples with internal standards is an alternative approach [83], with potential to improve the routine analysis of trace metals in a wider range of biological samples.

### Potential for single-cell analysis

LIBS has not been extensively applied to single-cell analysis but has been explored as a diagnostic instrument in the identification of microorganisms (bioaerosols, bacteria and their spores and fungi such as moulds and yeasts) [92]. This provides proof that LIBS can offer high spatial resolution in the 1–100-µm range for small sample sizes. Boyain-Goitia et al. [93] showed that LIBS could be used to analyse single pollen particles, providing proof of concept for the analysis of single particles. Nanosecond LIBS has been successfully used to discriminate between *Escherichia coli* strains, mould and the yeast *Candida albicans* [71]. Calcium, sodium and potassium have also been measured reproducibly in single fungal spores and bacteria demonstrating that LIBS could have the sensitivity to explore single cells [94]. Ng et al. [95] showed that LIBS (193 nm laser) was able to measure sodium and potassium in human red blood cells. A significant advance was shown by the work of Meng et al. [73], who successfully imaged the distribution of indium phosphide nanoparticles in lysosomes from freeze-dried macrophages using both confocal laser scanning microscopy and nanoscale LIBS. This is the first application of nanolaser-probed double-pulsed LIBS to visualise sub-cellular components [96], and demonstrates that this instrument has untapped potential in single-cell and sub-cellular imaging.

## LA-ICP-MS

LA-ICP-MS combines the LA process with conventional ICP-MS and provides an alternative approach to existing techniques where samples must be in the liquid phase and no spatial information is provided. LIBS overcomes a number of these disadvantages but does not have the same detection power and sensitivity as the ICP-MS system, and therefore, single-cell applications are challenging. LA-ICP-MS provides the benefit of increased sensitivity (ppb) with elemental mapping and high spatial resolution and requires little sample preparation [14]. As LA-ICP-MS tissue analysis has benefited from a wide range of research that has been covered in other publications [97, 98], this section will focus on calibration approaches for single-cell analysis and recent developments in this area.

### Calibration for single-cell analysis

LA-ICP-MS requires bespoke calibration approaches, and its lateral resolution ranges from 200 nm to 2 µm depending on the sample matrix, mass analyser, wavelength and type of laser being used [23]. Quantitative calibration based on matrix-matched standards is an attractive alternative and a key area of LA-ICP-MS analytical research. In one approach, a calibration curve of nanoparticle droplets deposited on a matrix-matched nitrocellulose membrane was used for the quantitative imaging of gold and silver in single fibroblast cells [99]. This provided high spatial resolution of intracellular patterns of the cytosol and perinuclear region. Nitrocellulose membrane–based standards have also been trialled for the visualisation and quantification of artificially introduced metals accumulated in the nuclei of single cells [100].

Dried droplets of gold chloride in water have been used as matrix-matched calibration standards for the quantification of gold nanoparticles in macrophages by adding rhodamine B (2% wt) to simulate the carbon content and improve visualisation of the cells [101]. A commercial inkjet printer was used to deposit the droplets of standards onto glass slides, which under controlled conditions offered ≤ 1% variation in deposited mass. The use of a Au solution for calibration was appropriate in this case as the 30-nm gold nanoparticles were laser-ablated particles of sufficiently small diameter to also behave as solutions in the ICP. Validation was carried out by comparing the concentration of gold found using solution ICP-MS with the average gold content found in 70 single cells using LA-ICP-MS.

High-density microarray gelatin standards have been used [20] to quantify copper in *Scrippsiella trochoidea* following exposure to transition metals. Gelatin is protein rich and has ionisation properties resembling the cellular matrix [102]. Uneven air-drying of droplets (“coffee ring effect”) needs to be considered; however, this can be overcome by sampling the whole spot*.* Alternatively, oven-dried gelatin droplet standards resulted in more homogenous elemental distribution [103]. Iodine has been used as an internal standard in tissue and accumulates in the cell nuclei, allowing cells and sub-cellular components to be imaged and inhomogeneities in tissue thickness to be normalised [104]. Elemental markers such as calcium, copper, zinc and carbon that correlate to cell volume have also been explored with limited success [105]. It was shown that anti-actin provided information on the structure and volume of cells, which could be applied for normalisation of signals.

LA-ICP-MS has many characteristics that make it ideal for single-cell analysis; however, it is still limited by slow throughput and a lack of suitable standards. The most promising development is the use of an alternative calibration approach known as single-cell isotope dilution [106], which uses a microfluidic device to deposit single cells in an array. Subsequently, each cell is dispensed with a droplet of an enriched isotope solution as a means of calibration by a commercial inkjet printer, followed by analysis using LA-ICP-MS. The single cells and isotope-enriched droplets are ablated simultaneously and mixed homogenously in the plasma. This promising approach both overcomes the need for commercial reference materials and improves the low analytical throughput.

### Recent developments and future research

Table 3 summarises the expansion of the applications of LA-ICP-MS by improving current methods for biological cell analysis with important developments in matrix-matched calibration standards. A very promising development in single-cell imaging has been the combination of micro-computed topography (µ-CT) and LA for three-dimensional single-cell imaging. Van Malderen et al. [107] described the use of metal staining to track the nuclei of human cervical carcinoma cells and combined this with information on the morphology of individual cells obtained using µ-CT.

**Table 3:** Examples of research publications in LA-ICP-MS analysis, detailing elements, sample type, laser used, quantitative/qualitative nature and calibration approach

Application	Elements	Sample	Laser (nm)	Quantitative/qualitative	Calibration	Reference
Bioimaging of metals in tissue	Au, C, Cu, Fe, Mg	Histological tissue of human eyes	213	Qualitative	N/A	[121]
	C, Cu, Ru, Y, Zn	Chicken breast tissue	213	Quantitative	PMMA spiked film standards	[122]
	Cu, Fe, K, Mg, Mn, Na, Ni, Zn	Human malignant pleural mesothelioma tissue	213	Quantitative	Dried droplet standards	[123]
	Fe	Sheep brain tissue	213	Quantitative	Frozen, homogenised slices of spiked tissue	[124]
	Ir	Mouse kidney tissue	193	Quantitative	Cellulose acetate–based spiked standards	[125]
Cellular applications	I	3T3 cells	213	Qualitative	N/A	[104]
	Dy, Eu, Ho, Ir, Lu Nd, Tb, Tm, Yb	3T3 cells	213	Quantitative	Spiked nitrocellulose membrane spots	[105]
	Cu	*S. trochoidea* cells	193	Quantitative	Microarray gelatin	[20]
	Ho, Ir, Tm	MDA-MB-231 X4 and MDA-MB-468 cells	193	Quantitative	Gelatin standards	[103]
	Ho, Ir	3T3 cells	213	Quantitative	Spiked nitrocellulose membrane spots	[108]
	Au, Cd	NIH/3T3 cells	266	Qualitative	N/A	[126]
	Au, Pt	HeLa cells	532	Qualitative	N/A	[127]
	Ag, Au	3T3 cells	213	Quantitative	Spiked nitrocellulose membrane spots	[99]
	Ir, Ln	3T3 cells, A549 cells	213	Quantitative	Spiked nitrocellulose membrane spots	[100]
	Au	RAW 264.7 cells	213	Quantitative	Dried droplet standards	[101]
	Ag	16HBE cells	213	Quantitative	Dried droplet standards	[128]
	Au	RAW 264.7 cells	213	Quantitative	Isotope dilution	[106]

One of the main limitations of the use of LA-ICP-MS is the low sample throughput. The work by Löhr et al. demonstrated that accurate and statistically significant results required the analysis of more than 400 cells [108]. Therefore, to increase the cell throughput, the research group developed a new technique based on a piezo-acoustic microarrayer that enables the delivery of single cells ready for laser ablation [109]. Note that although these studies by Van Malderen et al. [107] and Löhr et al. [109] used tagging techniques to allow the detection of molecular biomarkers as employed in LA-CyTOF-MS, they have opened the door to other applications and further research for endogenous elements.

Combining LIBS and LA-ICP-MS for the spatial analysis of biological samples shows great promise [25, 27]. LIBS allows measurement of bulk components that are hard to analyse using ICP-based techniques whereas ICP-MS can measure trace metals that are below LIBS limits of detection. Therefore, applying both of these complementary atomic spectroscopy techniques has enormous potential in single-cell analysis, as it can open routes for the development of improved validation strategies.

Another area of development is the combination of LA with SP-ICP-MS for the mapping and quantification of nanoparticles (NP) in biological tissues. SP-ICP-MS has demonstrated its capacity to detect and quantify a range of nanoparticles and determine their size distribution. The main drawback of this technique is that in the case of the detection of NP in solid samples, including biological tissues, it requires dissolution of the material, which may be prone to losses and inefficient extraction, and it could affect the integrity of the nanoparticles. An interesting approach has been developed by Metapari and co-workers coined as LA-SP-ICP-MS that was first applied for the mapping of gold nanoparticles in sunflower tissues cultivated under hydroponic conditions [110] and then further developed by the group [111–113]. The principle is based on the careful optimisation of the conditions of laser ablation (laser fluence, beam size and dwell time) to minimise nanoparticle degradation during the ablation process and reduce interferences from dissolved species. The combination LA and SP-ICP-MS allows adding spatial resolution to the characterisation of nanoparticles in biological tissues. Wang et al. [114] successfully applied this technique to achieve imaging of the in vivo degradation of silver nanoparticles injected in the spleen, liver and kidneys of mice. After demonstration of the viability of this approach, further development is required to increase the lateral resolution of the technique to achieve information about particle distribution at the cellular level. The dimensionality of the multimodal information generated by LA-SP-ICP-MS can be increased by the use of TOF mass analysers, which would enable “quasi-simultaneous” detection of a number of isotopes. Although this approach has not yet been applied to biological samples or tissues, the work by Holbrook et al. [115] for the detection of multi-elemental particles (MEPs) in sediments has provided proof of the feasibility of this approach to “fingerprint” metal nanoparticles and therefore provide an additional tool for the study of the toxicology of synthetic nanoparticles in biological systems.

## Challenges and priority recommendations

The elemental analysis of tissues and cells is a critical area of bioanalytical chemistry which is advancing at an impressive rate. At the forefront of these developments are SP-ICP-MS, LIBS and LA-ICP-MS. Sample preparation has been considerably overlooked in SP-ICP-MS literature, and there is a strong need to evaluate the effect of fixation approaches on elemental concentrations at the single-cell level. Important inconsistencies in transport efficiency calculations and calibration approaches are also highlighted in this review, stressing the need to readdress the direction of SP-ICP-MS research towards the standardisation of quantitative measurements. An interesting approach is to combine LIBS techniques with LA-ICP-MS for elemental imaging to develop strategies to improve quantitative validation as this approach would provide valuable spatial analysis of both endogenous trace and bulk components of tissue and cellular samples.

One of the future challenges for the advancement of atomic spectroscopy for the analysis of biological samples is the development of techniques that enable the generation of multimodal information. The progress of LA-SP-ICP-MS methodologies has provided a step forward in this direction by offering multidimensional information on the distribution, concentration and size of nanoparticles in biological tissues. The challenge is now how to improve the lateral resolution of current instruments and sensitivity of detectors to locate and quantify nanoparticles at cellular level. Clases and González de Vega [116] highlighted the need to develop multi-omic technologies that can generate an understanding at the metallomic/proteomic interface. In other words, it is desirable not only to detect and quantify metals and metalloids at the cellular level, but also necessary to identify their chemical speciation to unravel their biological role.

Single-cell proteomics (SCP) technologies are growing at pace [117], and therefore, parallel ICP-MS and SCP experiments could help to establish relationships between metals and proteins at the single-cell level. For a more direct association between metals and their speciation, hyphenation of conventional separation techniques such as liquid chromatography or capillary electrophoresis with ICP-MS could enable multi-omic analysis. However, these are currently hindered by the extremely high sensitivity that is required for speciation at cellular level. Researchers in microfluidics have partially addressed this topic by developing systems that concentrate and focus some of the metal species in cells prior to the introduction into the ICP-MS, although this only enables the analysis of groups of cells rather than analysis at cellular level [118], so the understanding of heterogeneity in a cellular population would be still limited.

The development of successful techniques for the imaging/mapping of metal species in biological tissues and cells goes through instrumental advances that enable hyphenation of techniques such as LA-ICP-MS with matrix-assisted laser desorption/ionisation mass spectrometry imaging (MALDI-MSI) for the co-location of proteins and their metal cofactors [119]. Another example of current work that could potentially facilitate the generation of elemental and molecular information in a single analysis is the system developed by Hoegg and co-workers [120] that combines a molecular ionisation source (CAM) with a liquid sample-atmospheric pressure glow discharge microplasma (LS-APGD) and ultra-high-resolution mass spectrometer. In parallel to instrumental advances, the development of new powerful software is required to handle and maximise the information generated by these multimodal analytical approaches. More research is needed to validate and standardise procedures that enable preparation of the samples (such as fixation, embedding, ashing and staining) to avoid delocalisation of the metal species in the tissues as highlighted above. The development of robust analytical methods such as those described here, and the collaboration of scientists across disciplines, are critical to answer fundamental biological questions.

## Declarations

This work is supported by the Doctoral College at the University of Surrey, England. Figure 1, Fig. 3 and Fig. 4 were created using Biorender.com.
